# Prognostic relevance of ALT-associated markers in liposarcoma: a comparative analysis

**DOI:** 10.1186/1471-2407-10-254

**Published:** 2010-06-03

**Authors:** Lorenza Venturini, Rosita Motta, Alessandro Gronchi, MariaGrazia Daidone, Nadia Zaffaroni

**Affiliations:** 1 Department of Experimental Oncology, Fondazione IRCCS Istituto Nazionale dei Tumori, Milan, Italy; 2 Department of Surgery, Fondazione IRCCS Istituto Nazionale dei Tumori, Milan, Italy

## Abstract

**Background:**

Most cancers maintain telomeres by activating telomerase but a significant minority, mainly of mesenchymal origin, utilize an alternative lengthening of telomeres (ALT) mechanism.

**Methods:**

In this study we comparatively analyzed the prognostic relevance of ALT in a monoinstitutional series of 85 liposarcoma patients as a function of the marker (ALT-associated promyelocytic leukemia bodies (APB) versus heterogeneous telomeres) used to classify the tumor.

**Results:**

Independently of the detection approach, ALT proved to be a prognostic discriminant of increased mortality, although the prognostic relevance of the two markers appeared at different follow-up intervals (at 10 years for APB and 15 years for telomeres).

**Conclusions:**

Overall, we confirmed ALT as an indicator of poor clinical outcome in this disease and provide the first evidence that the sensitivity of the ALT predictive power depends, at least in part, on the method used.

## Background

A hallmark of cancer cells is their limitless proliferative potential, which is sustained by the activation of a telomere maintenance mechanism (TMM) [1]. In a high percentage of human tumors (> 85%), proliferation-dependent telomere shortening is counterbalanced by the synthesis of telomeric DNA, which is catalyzed by telomerase [2]. However, in few cancers that lack telomerase, an alternative lengthening of telomeres (ALT) mechanism is used [3]. There may be more than one ALT mechanism, but in at least some ALT-positive human cancer cells telomere length is maintained by recombination-mediated replication of telomeric DNA [4].

Characteristics of ALT-positive tumor cells include an extreme heterogeneity of telomere length, with telomeres ranging from very short to extremely long within the same cell, as well as the presence of subnuclear structures termed ALT-associated promyelocytic leukemia (PML) bodies (APB), which contain telomeric DNA, telomere binding proteins and proteins involved in DNA recombination and replication [5]. Assays to detect telomere length and APB have been developed and alternatively used to screen human tumor specimens for the occurrence of ALT. Available results indicate that ALT is more common in tumors of mesenchymal and neuroepithelial origin, including osteosarcomas [6], soft tissue sarcomas [7] and glioblastoma multiforme [8], and that the presence of ALT has prognostic significance that depends on tumor type. Specifically, in liposarcoma ALT proved to be a strong prognostic discriminant of increased mortality [9], whereas in glioblastoma the presence of ALT was associated to a better patient survival [8], suggesting that the prognostic relevance of ALT presumably reflects the distinct set of genetic changes that are associated to the activation of ALT in a given tumor type.

In the present study, we comparatively analyzed the prognostic relevance of ALT in a monoinstitutional series of liposarcoma patients as a function of the characteristic (heterogenoeus telomeres versus APB presence) used to classify the tumor, with the final aim to identify the most suitable marker.

## Methods

### Study population

Samples from 85 liposarcomas, all from adult patients (36 women and 49 men; median age, 52 years; range, 18-91) treated with a curative intent at the *Istituto Nazionale Tumori *of Milan from December 1986 to November 2003 were available for TMM analysis (Additional file 1, Table S1). The specimens, which represent a subset of a larger case series already characterized for TMM (Costa *et al*, 2006), were consecutive with respect to the availability of frozen tissue and adequate clinicopathologic and follow-up information. Twenty-two patients presented with primary tumors and 63 with recurrent disease (59 local-regional recurrences and 4 metastases), and they underwent different surgical procedures according to disease presentation. The median follow-up for the entire group, as of December 2008, was 118 months. During the follow-up, 36 patients died for cancer-related causes (30 within 10 years, another 2 from 10 to 15 years, and 4 after 15 years). Postoperative treatment was given when there was a high risk of recurrence: 18 patients were submitted to radiotherapy, 8 to chemotherapy, and 5 to radio-chemotherapy according to the treatment protocols of the multidisciplinary Soft Tissue Sarcoma Group of the Institute.

This study was approved by the Institutional Review Board of the Institute, and all patients provided written informed consent to donate to the Institute the leftover tissue after diagnostic procedures.

### Detection of APB, telomere length and telomerase activity (TA)

Tumor tissue was sampled by a pathologist at the time of surgery and flash frozen. A fragment of about 100 mg was cut from each lesion and further subdivided for APB detection, DNA extraction (for telomere length assessment) and protein extraction (for TA assay). APB were assayed by combined PML immunofluorescence and telomere fluorescence *in situ *hybridization [10]. PML was detected with anti-PML mouse antibody (Dako Cytomation; Glostrup, Denmark) plus anti-mouse FITC-labeled goat antibody (Sigma; St. Louis, MO). Telomere fluorescence in situ hybrization (FISH) was performed by denaturing slides together with 5'labeled Cy3-(5'CCCTAA3')_3 _PNA probe (Applied Biosystems, Framingham, MA) for 3 min at 80°C and hybridizing for 3 hs at room temperature. Slides were washed and counterstained with 4'6-Diamino-2-phenylindole (DAPI). Images were captured on a Nikon Eclipse E600 fluorescence microscope using ACT-1 (Nikon, Tokyo, Japan) image analysis software and processed using Adobe Photoshop Image Reader 7.0 software. APB status was determined according to previously defined criteria: the presence of an APB was defined by the localization of a telomeric DNA focus within a nuclear PML body, sections were scored as APB+ if they contained APB in ≥0.5% of tumor cells and a tumor was considered ALT+ when at least one section was APB+. A set of criteria was used to determine the APB status of tumor section. An APB was considered to be present only when the telomeric DNA fluorescence within a PML body was more intense than that of telomeres, and a cell was not considered to contain APB if more than 25% of the co-localized foci occurred outside the nucleus. To avoid false negatives, at least 2,000 tumor nuclei were examined, and the assay was repeated in the presence of negative results.

Telomere length was assessed by pulsed-field gel electrophoresis as previously described [11]. ALT status was determined by calculating whether the mean, variance, and semi-interquartile range of the terminal restriction fragment (TRF) length distribution were greater than 16 kb, 1,000 kb^2^, and 4 kb, respectively. Tumors were classified as ALT+ when two of three or three of three of these criteria were met for unimodal or bimodal TRF length distributions, respectively. Statistical analysis of TRF length distributions was done with Telometric software [12].

TA was measured by the telomeric repeat amplification protocol (TRAP)[13], with the TRAPeze kit (Intergen, Oxford, UK) as outlined in Costa *et al*.[9].

### Data analysis

The agreement between APB and TRF data was assessed by kappa statistics. The clinical end point of the study was cancer-related survival, and the time of its occurrence was computed from the date of first diagnosis to the time of death, or censored at the date of the last recorded follow-up for living patients. Survival curves were estimated by means of the Kaplan-Meier product limit method [14], and the Cox proportional hazards model [15] was used to calculate the hazard ratios (HR) and their confidence interval (CI). SAS software (SAS Institutes, Inc., Cary, NC) was used to perform statistical calculations, and a two-sided *P *value ≤0.05 was considered statistically significant.

## Results and discussion

**Figure 1:**
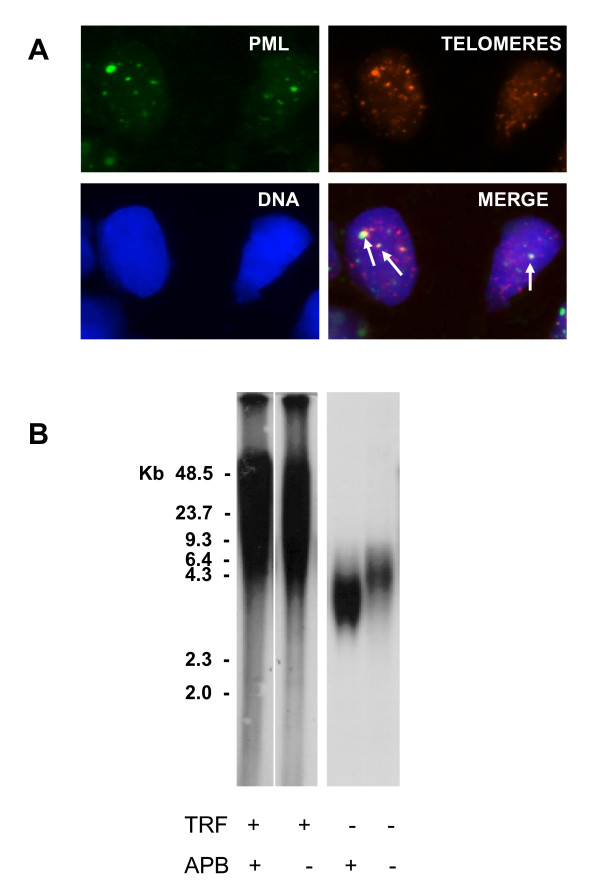
**ALT assays in liposarcomas**. **A) **APB assay: combined PML immunofluorescence and telomere fluorescence *in situ *hybridization (FISH) in a frozen section of an ABP-positive liposarcoma. Indirect immunofluorescence was used for the PML protein (FITC label, green stain). Telomere FISH was done using a Cy3-conjugated telomeric peptide nucleic acid probe (red stain). Nuclei were counterstained with 4',6-diamidino-2-phenylindole (blue stain). The foci of telomeric DNA that co-localize with PML represent APB. **B) **TRF southern blot analysis. Telomere length distribution of a representative series of liposarcomas. The lengths of telomeres in ALT-positive cells typically range from < 3 to > 50 kb. ALT-negative cells typically have a more homogeneous distribution of telomere length and a shorter average length than ALT-positive cells.

ALT status was determined on each liposarcoma specimen by using both APB detection and telomere length analysis (Fig. 1). Overall, 27 (31.8%) lesions were defined as ALT+ based on the presence of APB, whereas 24 (27.5%) samples were classified as ALT+ on the basis of TRF length distribution. A concordance between APB and TRF results in defining a specimen as ALT+ or ALT- was found in 66 of 85 cases (77.6%; kappa = 0.469; 95% CI, 0.265-0.672; *P *< 0.0001). Specifically, 16 lesions (18.8%) were defined as ALT+ and 50 (58.8%) were scored as ALT- with both detection methods. As regards the remaining lesions, 11 were defined as ALT+ on the basis of APB expression but did not show a TRF length distribution consistent with an ALT phenotype, and 8 were classified as ALT+ on the basis of TRF analysis but showed a very low percentage of APB-expressing tumor cells (from 0.01 to 0.2%).

The incomplete overlapping of the results obtained with the two methods is not surprising. In fact, while the APB assay allows the analysis of individual tumor cells, the TRF pattern could be misleading due to the admixture of normal and tumor cells present in the specimen. However, it has been recently shown by Jeyapalan *et al*. [16] that some telomerase-negative liposarcomas without APB express recombination-like activity at the telomere, suggesting that the incidence of ALT, as defined solely on the basis of APB expression, could be underestimated.

Thirty of 85 (35.3%) liposarcoma specimens were classified as TA+ based on positive TRAP results. Among these, 6 and 8 lesions were defined as ALT+/TA+ based on the expression of APB or on TRF length distribution, respectively, thus confirming the possibility that the two TMM coexist in the same lesion as previously reported also for other tumor types [3,17,18].

**Figure 2:**
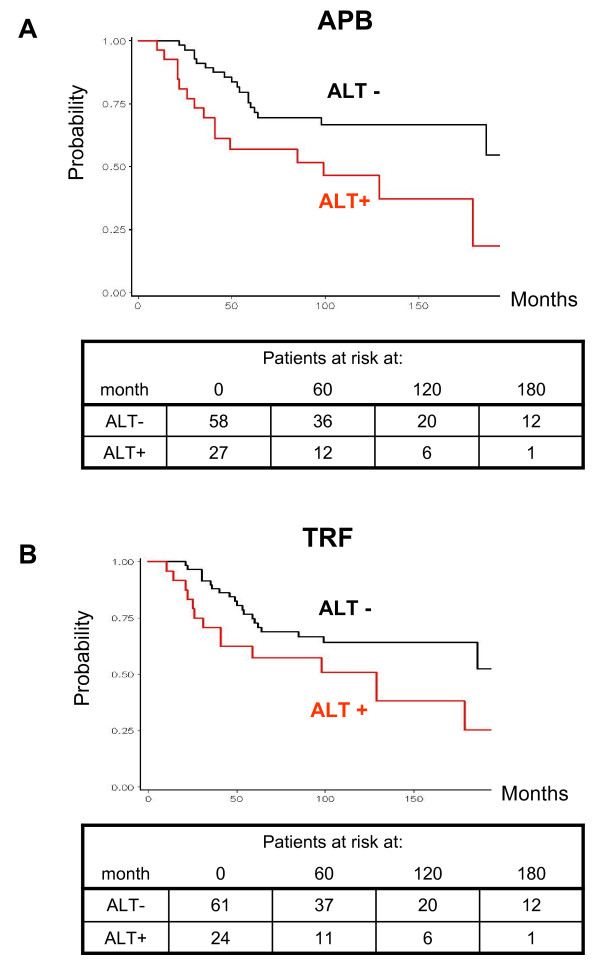
**Probability of disease-specific survival as a function of ALT, detected by APB presence (A) or TRF length distribution (B), according to the criteria reported in *Methods*, in liposarcoma patients**.

The prognostic significance of TMM was analyzed on the overall series of 85 patients. TA alone did not prove to be associated with disease-specific mortality (120 months: TA+ versus TA-, 62.0% versus 60.0%; HR, 0.91; 95% CI, 0.43-1.95; *P *= 0.814) (180 months: 62.0% versus 48.5%; HR, 0.80; 95% CI, 0.38-1.70; *P *= 0.566), whereas significant results were obtained for ALT. Specifically, when a tumor was defined as ALT+ according to at least one method (APB or TRF), ALT proved to be prognostic for 10-year disease-specific survival (ALT+ versus ALT-, 45.5% versus 71.1%; HR, 2.38; 95% CI, 1.15-4.90; *P *= 0.019), and such a prognostic value was maintained and strengthened at 15 years of follow-up (ALT+ versus ALT-, 25.3% versus 71.1%; HR, 2.76; 95% CI, 1.36-5.06; *P *= 0.005). These results held true also when APB expression was used as the only parameter to classify tumors for ALT. Specifically, the APB presence proved to be an indicator of increased mortality at both 10 years (HR, 2.14; 95% CI, 1.04-4.41; *P *= 0.040) and 15 years (HR, 2.54; 95% CI, 1.27-5.11; *P *= 0.009) of follow-up (Fig. 2A). Conversely, at 10 years of follow-up, patients with a tumor defined as ALT-positive on the basis of TRF length distribution showed a lower although not statistically significant probability of being alive (HR, 1.77; 95% CI, 0.84-3.73; *P *= 0.130). Such a trend reached statistical significance at 15 years (HR, 2.14; 95% CI, 1.06-4.34; *P *= 0.035) (Fig. 2B). These results held true even after adjustment for TA. In fact, the prognostic significance of APB expression was evident both at 10 (HR, 2.16; 95% CI, 1.03-4.51; *P *= 0.041) and at 15 years (HR, 2.53; 95% CI, 1.24-5.15; *P *= 0.011) of follow up, whereas TRF length distribution provided significant information only at 15 years of follow-up (HR, 2.11; 95% CI, 1.04-4.23; *P *= 0.038).

## Conclusions

In agreement with previously published results based on APB detection [9], we confirmed ALT as a prognostic discriminant of increased mortality in liposarcomas and provide the first evidence that sensitivity of the ALT predictive power depends, at least in part, on the marker (APB expression versus TRF length distribution) used. Notwithstanding the good agreement observed between the two assays in defining the ALT phenotype, they do not precisely identify the same subset of patients, conversely to that observed in glioblastoma multiforme, where a complete agreement in the results of the two assays was observed [10]. The incomplete overlapping of the TRF and APB results may be due to the different liposarcoma histological subtypes and this heterogeneity may result in a slight difference on the time-dependence of each assay to provide significant prognostic information. Overall, APB may be more appropriate than TRF pattern to assay ALT in tumors because they can be detected in both frozen and formalin-fixed, paraffin-embedded tumor samples - as we recently reported in this tumor type [19] - as well as in needle biopsies or cytology specimens. However, additional studies aimed at comparing the prognostic significance of results obtained with APB and TRF assays in other tumor types are warranted to provide reliable indications on the most appropriate ALT-related marker to be used for prognostic purposes.

## Abbreviations

ALT: Alternative lengthening of telomeres; TMM: Telomere maintenance mechanisms; APB: ALT-associated Promyelocitic leukaemia bodies; TRF: Terminal restriction fragment; TA: Telomerase activity; HR: Hazard ratio.

## Competing interests

The authors declare that they have no competing interests.

## Authors' contributions

LV carried out the molecular studies (APB detection) and drafted the manuscript. RM carried out the molecular studies (TRF analysis). AG provided tumour material and follow-up data

MGD participated in the design of the study, performed the statistical analysis and helped to draft the manuscript.

NZ conceived the study, partecipated in its coordination and helped to draft the manuscript. All the authors read and approved the final manuscript.

## Pre-publication history

The pre-publication history for this paper can be accessed here:

http://www.biomedcentral.com/1471-2407/10/254/prepub

## Supplementary Material

Additional file 1 **Table S1.** Patients and tumor characteristics. Click here for file
