# Supplementary material for: Prognostic relevance of ALT-associated markers in liposarcoma: a comparative analysis
Source: BMC Cancer. 2010 Jun 3;10:254. doi: 10.1186/1471-2407-10-254 (PMC2894794; doi:10.1186/1471-2407-10-254)
Supplement: Additional file 1 — Table S1. Patients and tumor characteristics. [file 1471-2407-10-254-S1.DOC]

Table S1: patients and tumors characteristics.

M= male, F= female, P= primary, R= local recurrence, M= metastasis, WD= well-differentiated, DE= dedifferentiated, MY= myxoid, RC= round-cell, PL= pleomorphic, CT= chemotherapy, RT= radiotherapy, DOD= dead of disease, DO= dead of other causes, AWD= alive with disease, NED= not evidence of disease, TA= telomerase activity, APB= ALT-associated promyelocytic leukaemia bodies, TRF= terminal restriction fragments.
